# Long-Term Efficacy of Subcutaneous C1 Inhibitor in Pediatric Patients with Hereditary Angioedema

**DOI:** 10.1089/ped.2020.1143

**Published:** 2020-09-16

**Authors:** Donald Levy, Teresa Caballero, Iftikhar Hussain, Avner Reshef, John Anderson, James Baker, Lawrence B. Schwartz, Marco Cicardi, Subhransu Prusty, Henrike Feuersenger, Ingo Pragst, Michael E. Manning

**Affiliations:** ^1^ University of California at Irvine, Irvine, California, USA.; ^2^ Hospital Universitario La Paz, Madrid, Spain.; ^3^ Vital Prospects Clinical Research Institute, Tulsa, Oklahoma, USA.; ^4^ Barzilai Medical Centre, Ashkelon, Israel.; ^5^ Clinical Research Center of Alabama, Birmingham, Alabama, USA.; ^6^ Baker Allergy, Asthma and Dermatology Research Center, Portland, Oregon, USA.; ^7^ Virginia Commonwealth University, Richmond, Virginia, USA.; ^8^ University of Milan, Milan, Italy.; ^9^ CSL Behring GmbH Standort Behringwerke Marburg, Marburg, Germany.; ^10^ Medical Research of Arizona, Scottadale, Arizona, USA.

**Keywords:** C1 inhibitor, COMPACT, hereditary angioedema, Haegarda, long term, safety, pediatric, children, prophylaxis, quality of life

## Abstract

***Background:*** Hereditary angioedema (HAE) due to C1 inhibitor (C1INH) deficiency is characterized by recurrent attacks of edema of the skin and mucosal tissues. Symptoms usually present during childhood (mean age at first attack, 10 years). Earlier symptom onset may predict a more severe disease course. Subcutaneous (SC) C1INH is indicated for routine prophylaxis to prevent HAE attacks in adolescents and adults. We analyzed the long-term efficacy of C1INH (SC) in subjects ≤17 years old treated in an open-label extension (OLE) of the pivotal phase III Clinical Study for Optimal Management of Preventing Angioedema with Low-Volume Subcutaneous C1 Inhibitor Replacement Therapy (COMPACT) trial.

***Methods:*** Eligible subjects (age ≥6 years, with ≥4 attacks over 2 consecutive months before entry into the OLE or placebo-controlled COMPACT trial) were treated with C1INH (SC) 40 or 60 IU/kg twice weekly for 52–140 weeks. Subgroup analyses by age (≤17 vs. >17 years) were performed for key efficacy endpoints.

***Results:*** Ten subjects were ≤17 years old [mean (range) age, 13.3 (8–16) years, 3 subjects <12 years old; exposure range, 51–133 weeks]. All 10 pediatric subjects experienced ≥50% reduction (mean, 93%) in number of attacks versus the prestudy period, with a 97% reduction in the median number of attacks/month (0.11). All subjects had <1 attack/4-week period and 4 had <1 attack/year (1 subject was attack free). No subject discontinued treatment due to a treatment-related adverse event.

***Conclusions:*** Data from pediatric subjects treated with C1INH (SC) for up to 2.55 years and adult subjects revealed similar efficacy. C1INH (SC) is effective and well tolerated as long-term prophylaxis in children, adolescents, and adults with HAE.

## Background

Hereditary angioedema (HAE) due to C1 inhibitor (C1INH) deficiency is an autosomal dominant disorder characterized by recurrent swelling episodes involving peripheral sites in the face, limbs, and trunk, as well as gastrointestinal, genitourinary, and/or laryngeal mucosal sites. Attacks may be disfiguring, disabling, and, in the case of laryngeal angioedema, life threatening.^1–3^ Abdominal attacks are characterized by intense pain, sometimes resembling a surgical abdomen, and may be accompanied by vomiting, diarrhea, and in severe cases, intestinal obstruction and loss of consciousness.^4^

Onset of attacks of HAE may occur in early childhood.^2,5,6^ The age of symptom onset ranges from 4.4 to 18 years, with a mean age at first attack of 10 years.^5^ In a large survey of 581 patients with HAE whose symptoms began before age 21,^6^ earlier onset of symptoms was associated with a more severe disease course,^6^ including a greater number of attacks per year and greater number of hospitalizations due to HAE signs and symptoms.^6^ Patients with symptom onset at an earlier age also reported a greater negative impact on their quality of life (QoL).^6^ In a study of 34 pediatric patients with HAE (mean age, 12.3 years), symptomatic children reported impaired QoL compared with healthy controls or children with an asymptomatic disease course, particularly with respect to school performance and physical domain metrics.^7^ More severe disease activity was associated with greater QoL impairment. Children with ≥5 attacks per year reported greater impairment in QoL than those with <5 attacks per year.^7^

Prophylactic therapy for HAE aims to reduce the frequency and severity of attacks, with the overall goal of improving QoL.^8^ The 2017 World Allergy Organization (WAO) guidelines for the treatment of HAE have recommended plasma-derived (pd) C1INH as the preferred therapy for on-demand treatment and long-term prophylaxis in children based on its efficacy, tolerability, and good safety profile in pediatric patients.^8^ International consensus guidelines from the Hereditary Angioedema International Working Group in 2017 also state that pdC1INH may be the safest approach for long-term prophylaxis in children.^5^

Intravenously administered pdC1INH formulations are available for on-demand treatment and routine prophylaxis in pediatric patients with HAE, including children <12 years of age.^9,10^ However, administration of intravenous (IV) C1INH for long-term prophylaxis requires repeated venous access, which may be difficult to sustain over time, and may lead to local and systemic complications.^11^ Subcutaneous (SC) administration of C1INH prophylactic therapy can provide a convenient alternative to IV therapy for younger patients and their caregivers.^12^

A C1INH formulation administered subcutaneously [C1INH (SC) 60 IU/kg, HAEGARDA^®^; CSL Behring, Marburg, Germany] was approved by the US Food and Drug Administration (FDA) in June 2017 as routine prophylaxis to prevent HAE attacks in adolescents and adults.^12–15^ Approval was based on the results of the COMPACT (Clinical Study for Optimal Management of Preventing Angioedema with Low-Volume Subcutaneous C1 Inhibitor Replacement Therapy) study program.^12,14–16^ In the pivotal phase III, placebo-controlled, crossover COMPACT trial, the FDA-approved 60 IU/kg dose of C1INH (SC) administered twice weekly resulted in a 95% median reduction in attacks relative to placebo, as well as a 100% median reduction in the use of on-demand medications.^16^ The long-term safety and efficacy of C1INH (SC) were further evaluated in an open-label extension (OLE) of the COMPACT trial in which subjects were treated for up to 140 weeks (2.7 years).^17^ In the OLE study, C1INH (SC) was well tolerated and had a marked and sustained effect, with a median attack rate of 1.0 attack per year in subjects treated with the 60 IU/kg dose.^17^

In this analysis, we evaluated the long-term efficacy and safety of C1INH (SC) in 10 pediatric subjects (7 adolescents 12–17 years old and 3 children <12 years old) treated in the OLE of the COMPACT trial, and compared them with adult subjects (>17 years) in the OLE population.

## Methods

### COMPACT OLE study design

**FIG. 1.**
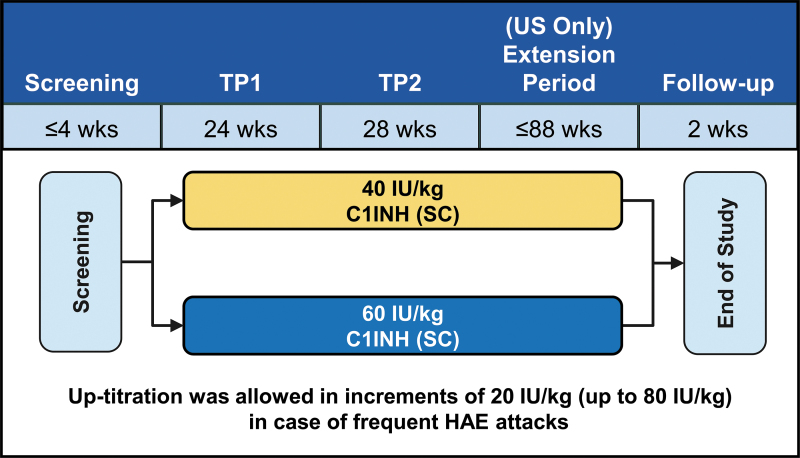
COMPACT OLE study design. COMPACT, Clinical Study for Optimal Management of Preventing Angioedema with Low-Volume Subcutaneous C1 Inhibitor Replacement Therapy; OLE, open-label extension; TP, treatment period.

The OLE of the COMPACT trial was a multicenter, randomized, parallel-arm study and enrolled patients treated in the placebo-controlled COMPACT trial, as well as C1INH (SC)-naive patients.^17^ Eligible patients (age ≥6 years with at least 4 attacks over 2 consecutive months before enrollment in the OLE or the placebo-controlled COMPACT trial) were randomly assigned to receive C1INH (SC) 40 or 60 IU/kg twice weekly for 52 weeks.^17^ Subjects in the United States could continue therapy for an additional 88 weeks (maximum exposure of 140 weeks) (Fig. 1). Dose increases in increments of 20 IU/kg (up to 80 IU/kg) were permitted at the investigator's discretion in case of frequent HAE attacks.^17^

The primary objective of the OLE was to assess the long-term safety of C1INH (SC).^17^ The secondary efficacy endpoints were the percentage of responders (≥50% reduction in attacks compared with the prestudy period) and percentage of subjects with <1 attack per 4-week period.^17^ Subgroup analyses by age (≤17 vs. >17 years at study entry) were performed for these 2 secondary endpoints, as well as the time-normalized number of HAE attacks (exploratory endpoint). Use of on-demand medication was evaluated in a *post hoc* analysis.

The evaluation period for the efficacy analysis began on day 1 of week 3 of treatment. HAE attacks that occurred within the first 2 weeks after starting treatment with C1INH (SC) were not counted in the efficacy analysis because this was a prespecified wash-in period to enable attainment of steady-state C1INH levels.

Subjects recorded their HAE symptoms daily in an electronic diary (eDiary), including the time of onset, anatomic location, severity, and use of on-demand medication for treatment of attacks. At each study visit, the investigator reviewed the subject eDiary and recorded the stop/start dates, anatomic location, and severity of HAE attacks in the electronic case report form.

Subjects self-assessed their response to treatment every 6 months using the Subject's Global Assessment of Response to Treatment (SGART) (0 = none; 1 = poor; 2 = fair; 3 = good; and 4 = excellent). The SGART was an exploratory efficacy endpoint. Response to C1INH (SC) prophylaxis was also evaluated by the investigator using the Investigator's Global Assessment of Response to Therapy (IGART). The IGART required the investigator to rate each subject's response to treatment using the same scale as the SGART.

The OLE study (NCT02316353) on which this subgroup analysis is based was done in accordance with the standards of Good Clinical Practice as defined by the International Council for Harmonization of Technical Requirements for Registration of Pharmaceuticals for Human Use, ethical principles that have their origin in the Declaration of Helsinki, and applicable national and local regulations. Study Protocol and amendments were approved by independent ethics committees or institutional review boards at all participating centers before study commencement. All patients, or their legal guardians, provided written informed consent.

## Results

A total of 126 subjects were randomly assigned to treatment with C1INH (SC), 63 to each dose group (40 and 60 IU/kg). Ten subjects were ≤17 years old [mean age, 13.3 years (range, 8–16 years)]; of these, 7 were adolescents between the ages of 13 and 16, and 3 were children between the ages of 8 and 10; 4 were female and 6 were male.

Among the 10 subjects, 5 had been previously treated with C1INH (SC) in the COMPACT trial and 5 were C1INH (SC) naive. In the OLE, 5 subjects were treated with the 40 IU/kg dose and 5 were treated with 60 IU/kg. No subject required a dose increase to optimize treatment. The mean duration of exposure to C1INH (SC) among the pediatric subjects was 89 weeks (range: 51–133 weeks).

### Efficacy outcomes

**FIG. 2.**
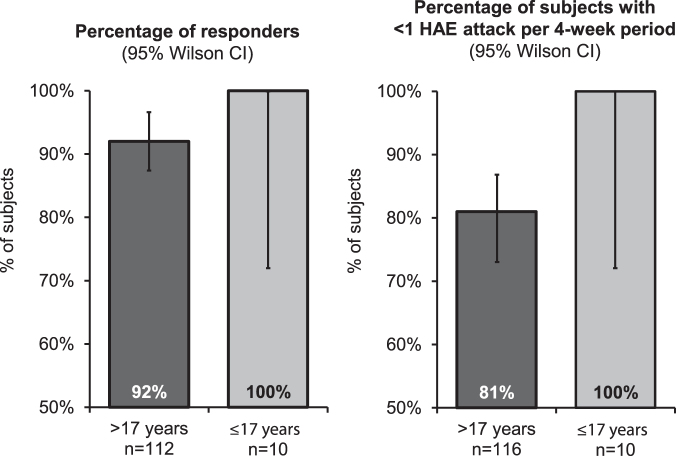
Efficacy in pediatric and adult subjects treated long term with C1INH (SC). C1INH (SC), subcutaneous C1 inhibitor.

All 10 (100%) subjects ≤17 years old and 103 of 112 (92%) evaluable subjects >17 years old were classified as responders, with ≥50% reduction in attacks compared with the prestudy period. Similarly, all 10 (100%) subjects ≤17 years old and 94 of 116 (81%) subjects >17 years old experienced <1 HAE attack per 4-week period during C1INH (SC) treatment (Fig. 2).

All subjects ≤17 years old experienced substantial reductions in the number of attacks per month with C1INH (SC) prophylaxis, with the percentage reduction in HAE attacks per month ranging from 76% to 100%. Four subjects had <1 attack per year during prophylaxis with C1INH (SC), and 1 pediatric subject (age, 10 years old) was attack free during the entire period of prophylaxis (1 year) (Table 1).

**Table 1.** Efficacy Outcomes in Pediatric Subjects (Age ≤17 Years) Treated Long Term with Subcutaneous C1 Inhibitor

Subject	Age at study entry (years)	C1INH (SC) treatment status	C1INH (SC) dose at randomization (IU/kg)	Duration of C1INH (SC) exposure in efficacy evaluation (weeks)^a^	Attack rate with C1INH (SC) prophylaxis (attacks/month)	% reduction in attacks/month relative to prestudy period
1	8	Naïve	40	50	0.09	97
2	10	Naïve	60	51	0	100
3	10	Naïve	40	120	0.04	96
4	13	Naïve	60	50	0.26	96
5	14	Previously treated	40	126	0.24	93
6	15	Previously treated	60	49	0.79	76
7	15	Previously treated	40	128	0.14	97
8	16	Previously treated	40	52	0.92	77
9	16	Previously treated	60	131	0.03	98
10	16	Naive	60	114	0.04	99

^a^ The first 2 weeks of treatment were excluded from the efficacy analyses.

C1INH (SC), subcutaneous C1 inhibitor.

Outcomes in subjects ≤17 years old were generally similar to those observed in subjects >17 years old. The median number of HAE attacks per month was 0.11 in subjects ≤17 years old and 0.09 in subjects >17 years old; the median percentage reduction in attacks was 97% and 98%, respectively. Mean rescue medication use was 0.09 uses/month among subjects ≤17 years old and 0.31 uses/month among subjects >17 years old.

Among the pediatric subjects treated, a total of 38 attacks occurred during the treatment evaluation period. The most common anatomic locations of the attacks were the abdomen (20 attacks) and extremities (13 attacks). One pediatric subject on the 40 IU/kg dose experienced a mild laryngeal attack. Four attacks involved multiple locations. Of the 38 attacks, 25 were considered mild, 9 were of moderate severity, and 4 were severe. In addition, 16 (42%) of the 38 attacks were treated with rescue medications (8, mild; 4, moderate; and 4, severe). All 4 severe attacks that were treated had abdominal involvement.

### Safety outcomes

In the overall population, as well as in the pediatric subgroup (*n* = 10), C1INH (SC) was well tolerated; injection-site reactions were the most common adverse event (AE). In the pediatric subgroup, injection-site erythema was the most common treatment-related AE and was reported by 2 subjects (Table 2). Notably, 7 of the 10 pediatric subjects did not report any injection-site reactions. Among the pediatric subjects, all injection-site reactions resolved, and all but 1 case of injection-site erythema were mild.

**Table 2.** Safety Profile of Subcutaneous C1 Inhibitor in Pediatric Subjects (Age ≤17 Years)

Subject	Age at study entry (years)	C1INH (SC) dose (IU/kg)	C1INH (SC) exposure (weeks)	Treatment-related AEs
1	8	40	52	Injection-site urticaria, injection-site pain, injection-site papule
2	10	60	53	None
3	10	40	122	None
4	13	60	52	None
5	14	40	128	None
6	15	60	51	None
7	15	40	130	Injection-site erythema
8	16	40	54	None
9	16	60	133	Injection-site erythema, injection-site induration
10	16	60	116	None

AE, adverse event; C1INH (SC), subcutaneous C1 inhibitor.

No pediatric subject discontinued treatment due to a treatment-related AE. There were no reports of serious AEs, thromboembolism, or anaphylaxis in the pediatric subgroup.

### Subject and investigator assessments of response to therapy

A total of 9 of 10 pediatric subjects self-assessed their response to treatment as excellent (SGART score = 4) at all assessment points. One pediatric subject (Subject 6) assessed his response to treatment as good (SGART score = 3) at the first assessment and excellent at the second. For all 10 pediatric subjects, the response to treatment was rated as excellent (IGART score = 4) by the respective investigators.

## Discussion

pdC1INH is the recommended therapy for long-term prophylaxis in pediatric and adult patients as per the 2017 WAO guidelines for the management of HAE.^8^ Moreover, based on the international consensus guidelines for the treatment of HAE in pediatric patients, pdC1INH is considered the safest prophylactic treatment and is recommended over attenuated androgens when possible.^5^ In this pediatric subgroup analysis of an open-label trial, C1INH (SC) was highly effective, with all subjects experiencing a ≥50% reduction in attacks compared with prestudy values, and 4 of 10 subjects experiencing <1 attack per year during prophylaxis. Data from pediatric subjects treated with C1INH (SC) for up to 2.55 years did not reveal any difference in efficacy compared with adult subjects. The median reduction in attacks relative to the prestudy period was 97%, similar to the 98% reduction observed in subjects >17 years old. The median number of attacks per month with C1INH (SC) prophylaxis in the pediatric subgroup was 0.11, or ∼1 attack per year, which again is similar to the median number observed in those >17 years old (0.09 attacks/month).

Eight of 10 patients achieved >90% reduction in attacks relative to the prestudy period, while Subjects 6 and 8 had a 76% and 77% reduction in attack frequency relative to the prestudy period, respectively (Table 2). Assessments of steady-state C1INH functional activity were performed at weeks 9, 25, 29, 37, and 53 during treatment. For Subject 6, C1INH functional activity was demonstrated to be below the critical threshold of 40% on week 29 and 37 assessments (12% and 25.1%, respectively). Steady-state C1INH functional activity for Subject 8 was barely below 40% at weeks 29 and 37 (39.8% and 39%, respectively) with all other measurements being >40%. While certain triggers are known to precipitate attacks in HAE patients (stress, trauma, infection, menstruation, and medications), many attacks occur without a known trigger, highlighting the unpredictable nature of HAE.^18^

In a *post hoc* analysis of all patients randomized in the OLE (*n* = 90), Lumry et al. reported on the benefit of C1INH (SC) of various patient-reported outcome QoL measures related to the burden of HAE such as clinically meaningful improvements in anxiety and depression scores, improved work productivity, and less nonwork activity impairment, as well as high treatment satisfaction and perceived effectiveness compared with on-demand treatment alone.^19^

The frequency, severity, and location of attacks are major factors associated with negative QoL in children with HAE.^7^ The inability to attend school and decreased involvement in social activities, as well as physical impairment result in reduced QoL of pediatric patients.^7^ Given that greater attack frequency is associated with reduced QoL, treatment with C1INH (SC) may have a significant impact on various aspects of pediatric patients physical, social, and emotional well-being.^7^ A subgroup analysis of pediatric patients from the OLE is underway.

Moreover, C1INH (SC) was safe and well tolerated in pediatric subjects with HAE. Only 3 of 10 subjects reported injection-site reactions associated with administration. All injection-site reactions resolved (>90% in 1–2 days), and the vast majority were mild. There were no reports of safety issues or serious AEs in this pediatric subgroup. As in the adult study population, there were no reports of anaphylaxis or related thromboembolic events. None of the pediatric subjects discontinued treatment due to an AE. Overall, pediatric subjects self-assessed their response to treatment as excellent at nearly all time points.

pdC1INH administered intravenously has been shown to be effective and well tolerated in the prophylactic treatment of HAE in children, as well as in adolescents and adults.^12,20–24^ In addition, prophylactic therapy with IV pdC1INH has been found to improve QoL in pediatric patients with HAE, especially at higher doses.^25,26^ SC injection of C1INH therapy should make administration easier, especially for adolescent and pediatric patients and their parents/caregivers.^12^ SC administration also facilitates the delivery of high concentrations of C1INH with low volumes of injection. Furthermore, C1INH (SC) 60 IU/kg provides steady-state C1INH levels and function near the low-normal range, with C1INH functional activity consistently maintained above 40%, a level associated with prevention of HAE attacks.^12^

As in the adult population, the most common types of attacks in the pediatric subgroup were abdominal or peripheral.^2^ Laryngeal attacks are of particular concern in young children with HAE, as the laryngeal airway is narrower and physiologic reserve is reduced compared with adults.^27,28^ In this group of 10 pediatric subjects treated with C1INH (SC) for up to 2.55 years, only 1 (mild) laryngeal attack was reported in a subject treated with the lower dose (40 IU/kg), while none of the pediatric subjects treated with the 60 IU/kg dose experienced a laryngeal attack.

The approved dose of C1INH (SC) in the United States is 60 IU/kg.^14^ Weight-based dosing facilitates individualization of therapy and enables clinicians to treat younger patients with appropriate doses of C1INH. A population pharmacokinetic analysis from the placebo-controlled COMPACT trial found that twice-weekly dosing with C1INH (SC) 60 IU/kg resulted in similar C1INH functional activity in pediatric and adult patients, with C1INH functional levels maintained above 50% in both groups.^29^

In summary, frequent attacks in pediatric patients with HAE can present a considerable burden, and lead to significant impairment in QoL, particularly with respect to school and physical activities.^7^ Initiation of prophylaxis in children and adolescents with HAE can reduce the burden of disease activity, thereby improving outcomes important to patients. Subcutaneously administered C1INH is an effective and well-tolerated option for long-term prophylaxis in pediatric patients with HAE.
